# Supplementary material for: Unifying machine learning and quantum chemistry with a deep neural network for molecular wavefunctions
Source: Nat Commun. 2019 Nov 15;10:5024. doi: 10.1038/s41467-019-12875-2 (PMC6858523; doi:10.1038/s41467-019-12875-2)
Supplement: Supplementary file 1 — Supplementary Information [file 41467_2019_12875_MOESM1_ESM.pdf]

Supplementary Information: Unifying machine learning and quantum chemistry with a deep neural network for molecular wavefunctions

Schütt et al.

Supplementary Table 1: **Overview of training settings for each data set.** The number of references calculations of the training, validation and test sets are given as well as the size of the mini batch n_{batch}, the initial learning rate lr_{init} and the number of epochs $t_{patience}$ without improvement of the validation error before the learning rate was decreased.

Data set	n_{train}	$n_{validate}$	n_{test}	n_{batch}	lr_{init}	$t_{patience}$
H_2O	500	500	4000	32	$1 \cdot 10^{-4}$	50
Ethanol (HF / DFT)	25000	500	4500	32	$1 \cdot 10^{-4}$	15
Malondialdehyde	25000	500	1478	32	$1 \cdot 10^{-4}$	15
Uracil	25000	500	4500	48	$1 \cdot 10^{-4}$	15

Supplementary Table 2: **Predictions errors of SchNOrb.** Mean absolute errors for the Hamiltonians, overlaps, energies ϵ and coefficients of occupied MOs as well as the total energy are given.

Data set	level of theory	basis set	H [eV]	S	ϵ [eV]	ψ	total energy [eV]
H_2O	DFT	def2svp	0.0045	7.91e-05	0.0076	1.00	0.001435
Ethanol	HF	def2svp	0.0079	7.50e-05	0.0106	1.00	0.000378
Ethanol	DFT	def2svp	0.0051	6.78e-05	0.0091	1.00	0.000361
Ethanol	DFT	def2tzvp	0.0083	9.59e-05	0.4775	0.70	*0.001533
Malondialdehyde	DFT	def2svp	0.0052	6.73e-05	0.0109	0.99	0.000353
Uracil	DFT	def2svp	0.0062	8.24e-05	0.0479	0.90	0.000848

* trained without atomic forces

Supplementary Table 3: **Errors for Loewdin population analysis.** Mean absolute errors for the predicted partial charges and bond orders.

Dataset	level of theory	basis set	partial charges	bond orders
Water	DFT	def2svp	0.002	0.001
Ethanol	DFT	def2svp	0.002	0.001
Ethanol	HF	def2svp	0.002	0.001
Malondialdehyde	DFT	def2svp	0.004	0.001
Uracil	DFT	def2svp	0.047	0.006

Supplementary Table 4: **Errors for molecular properties.** Mean absolute errors of the dipole and quadrupole moments computed for the predicted wavefunction coefficients.

Dataset	level of theory	basis set	dipole moment [D]	quadrupole moment [D Å]
Water	DFT	def2svp	0.0072	0.0061
Ethanol	DFT	def2svp	0.0262	0.0355
Ethanol	HF	def2svp	0.0161	0.0218
Malondialdehyde	DFT	def2svp	0.0536	0.0575
Uracil	DFT	def2svp	1.2762	1.8703

Supplementary Table 5: **Errors for MP2 energies.** Mean absolute errors of the Hartree-Fock energy, MP2 correlation energy and total MP2 energy (HF + MP2 correlation) computed for the HF ethanol dataset based on the predicted wavefunction coefficients. In addition, MAEs relative to the average energy values are provided in percent.

Energy component	MAE [eV]	MAE [%]
HF	0.0214	0.01
MP2 correlation	0.0831	17.19
HF + MP2	0.0926	0.06

Supplementary Table 6: **Duration of dataset generation as well as training and prediction using SchNOrb.** Each model was trained using 25,000 training examples on a Nvidia P100 GPU. The uracil model was trained on two P100 using data parallelism.

Data set	reference calculations [h]	training [h]	ML prediction [s]	QC calculation [s]	speed-up
Ethanol (HF)	65	87	0.033	9.1	276x
Ethanol (DFT)	157	82	0.033	22.2	673x
Malondialdehyde	309	71	0.034	43.6	1282x
Uracil	626	80	0.038	88.4	2326x

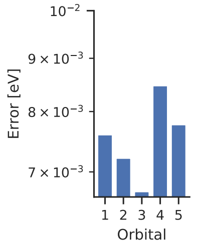

(a) H_2O, DFT

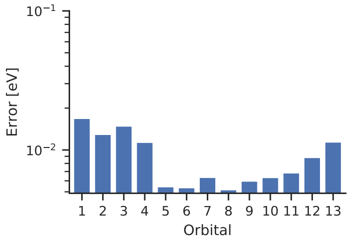

(b) Ethanol, DFT

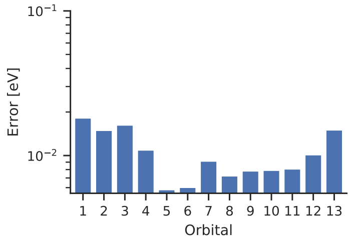

(c) Ethanol, Hartree-Fock

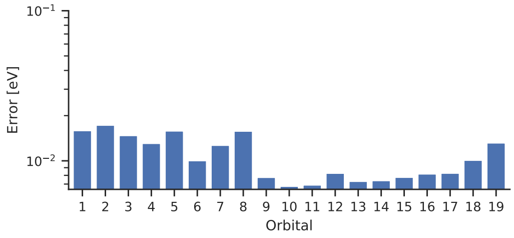

(d) Malonaldehyde, DFT

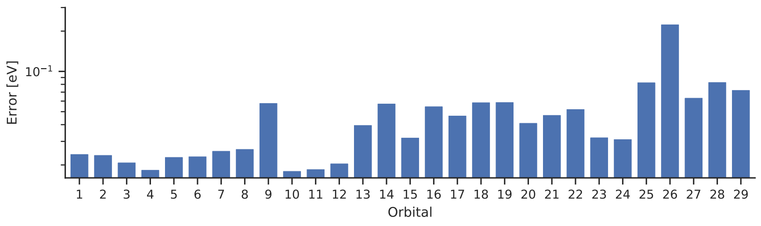

(e) Uracil, DFT

Supplementary Figure 1: **Mean absolute errors of energies separate for each occupied MO. (a) H_2O, (b) Ethanol, DFT, (c) Ethanol, Hartree-Fock, (d) Malonaldehyde, DFT, (e) Uracil, DFT .**

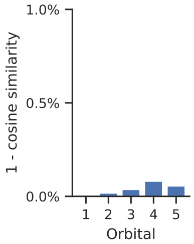

(a) H_2O, DFT

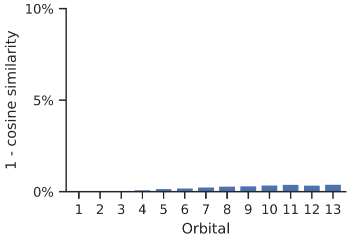

(b) Ethanol, DFT

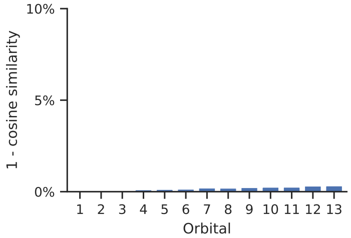

(c) Ethanol, Hartree-Fock

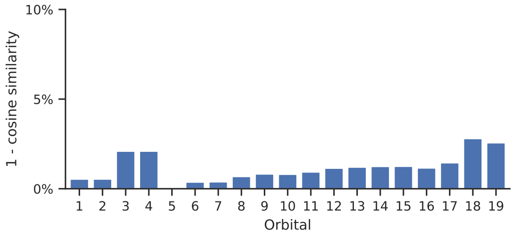

(d) Malonaldehyde, DFT

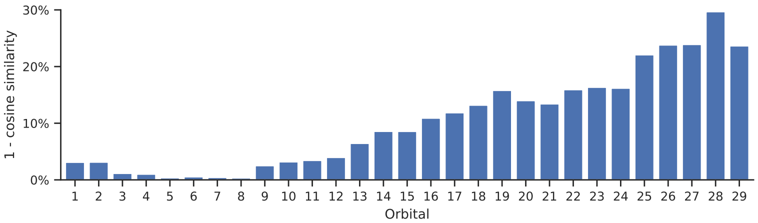

(e) Uracil, DFT

Supplementary Figure 2: **Mean cosine distances of coefficient vectors separate for each occupied MO.** The given cosine distances are equivalent to $1-$ cosine similarity. (a) H_2O, (b) Ethanol, DFT, (c) Ethanol, Hartree-Fock, (d) Malonaldehyde, DFT, (e) Uracil, DFT.

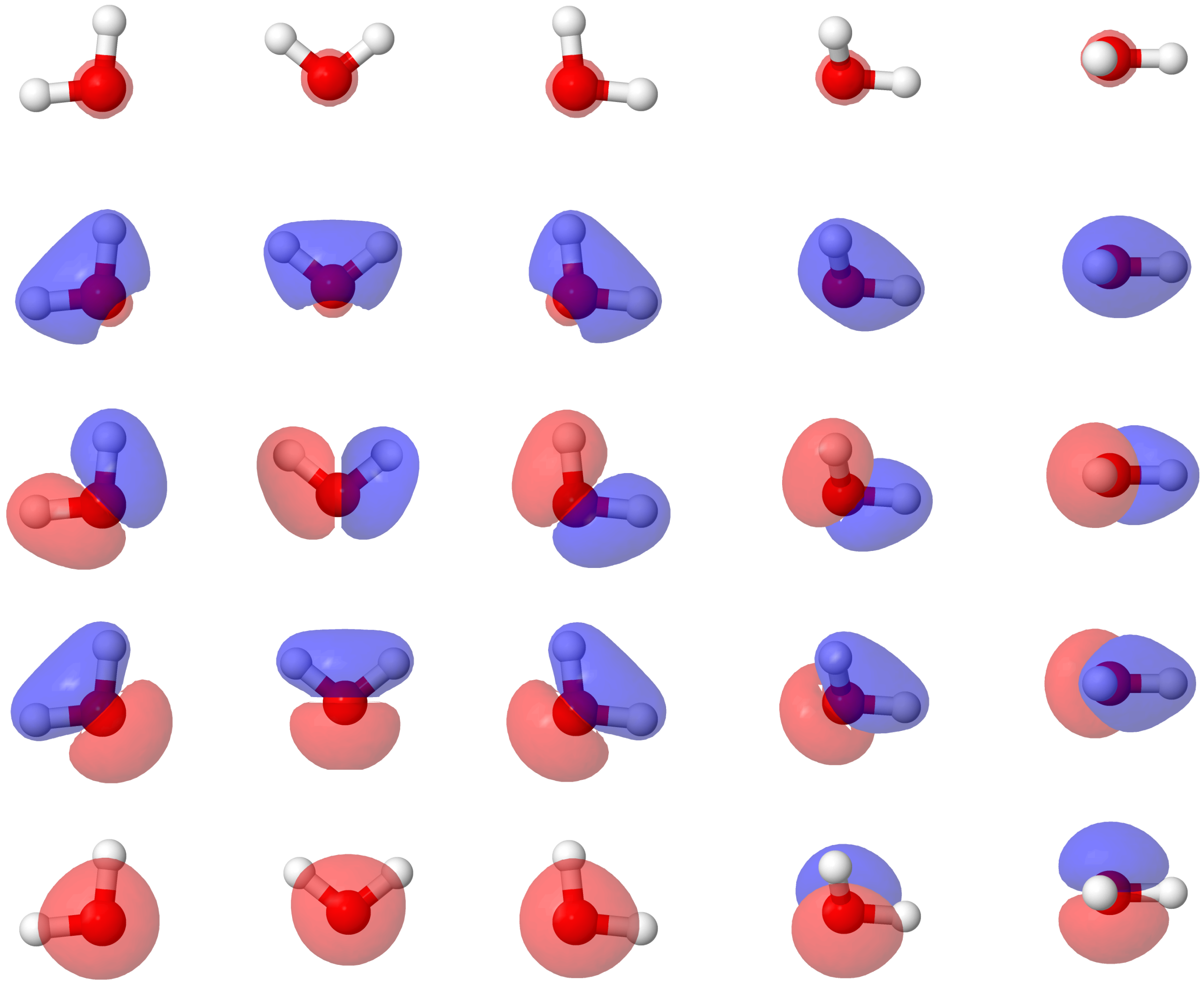

Supplementary Figure 3: **Rotations of H_2O and its predicted MOs.** Each row corresponds to an MO. The learned orbitals are covariant under rotation of the molecule.

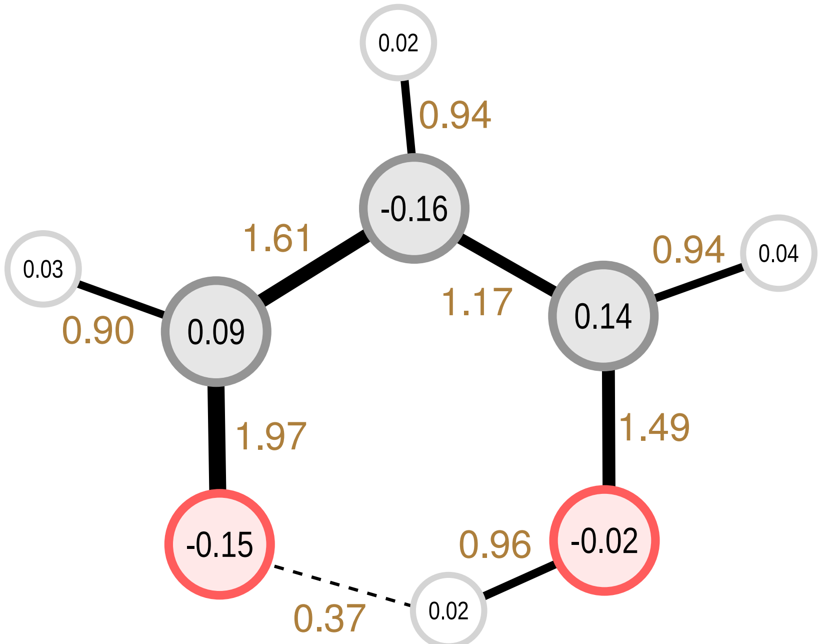

Supplementary Figure 4: **Loewdin population analysis for malondialdehyde.** The SchNOrb predictions of partial charges and bond orders for a randomly selected configuration of malondialdehyde are shown.

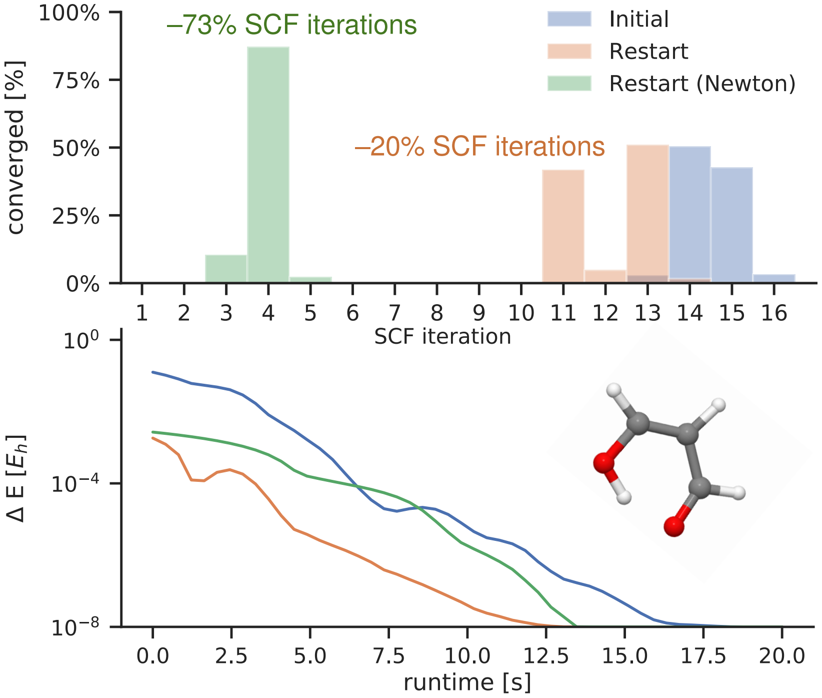

Supplementary Figure 5: **Accelerated SCF convergence of malondialdehyde.** The predicted MO coefficients for the malondialdehyde configurations from the test set are used as a wave function guess to obtain accurate solutions from DFT. (top panel) Histogram of SCF calculations vs. number of SCF iterations shows that this reduces the required SCF iterations by 73%. (bottom panel) Logarithmic energy change per SCF step vs. runtime in seconds shows that it is more efficient to use the conventional SOSCF method, even though this saves only 20% of iterations for malondialdehyde.
