# Supplementary material for: Unifying machine learning and quantum chemistry with a deep neural network for molecular wavefunctions
Source: Nat Commun. 2019 Nov 15;10:5024. doi: 10.1038/s41467-019-12875-2 (PMC6858523; doi:10.1038/s41467-019-12875-2)
Supplement: Supplementary file 3 — Description of Additional Supplementary Files [file 41467_2019_12875_MOESM3_ESM.docx]

**Description of Supplementary Files**

**File Name:** Supplementary Video 1

**Description:** The video shows a Kohn-Sham eigenstate during a molecular dynamics simulation for malondialdehyde. (left) the SchNOrb predicted wave function; (right) the original wave function as calculated by Density Functional Theory.
